# Compliance with referrals to medical specialist care: patient and general practice determinants: a cross-sectional study

**DOI:** 10.1186/s12875-016-0401-7

**Published:** 2016-02-01

**Authors:** Christel E. van Dijk, Judith D. de Jong, Robert A. Verheij, Tessa Jansen, Joke C. Korevaar, Dinny H. de Bakker

**Affiliations:** NIVEL, Netherlands Institute for Health Services Research, P.O.Box 1568, , 3500 BN, Utrecht, The Netherlands

**Keywords:** Referral and consultation, Primary health care, Patient compliance, Cross-sectional studies, Netherlands

## Abstract

**Background:**

In a gatekeeper system, primary care physicians and patients jointly decide whether or not medical specialist care is needed. However, it is the patient who decides to actually use the referral. Referral non-compliance could delay diagnosis and treatment. The objective of this study was to assess patient compliance with a referral to medical specialist care and identify patient and practice characteristics that are associated with it.

**Methods:**

Observational study using data on 48,784 referrals to medical specialist care derived from electronic medical records of 58 general practices for the period 2008–2010. Referral compliance was based on claims data of medical specialist care. Logistic multilevel regression analyses were conducted to determine associations between patient and general practice characteristics and referral compliance.

**Results:**

In 86.6 % of the referrals, patients complied. Patient and not practice characteristics were significantly associated with compliance. Patients from deprived urban areas and patients aged 18–44 years were less likely to comply, whereas patients aged 65 years and older were more likely to comply.

**Conclusion:**

About 1 in 8 patients do not use their referral. These patients may not receive adequate care. Demographic and socio-economic factors appear to affect compliance. The results of this study may be used to make general practitioners more aware that some patients are more likely to be noncompliant with referrals.

## Background

In many Western countries and health plans in the United States, patients have a primary care physician who acts as a formal gatekeeper for medical specialist care and thereby determines together with the patient whether or not a patient requires medical specialist care [1, 2]. Ideally in such systems, patients are treated in primary care if possible, and referred to medical specialist care if necessary. For the gatekeeper system to be effective, it is vital that adequate decisions are made about when and who to refer. But it is also important that referred patients comply with the referral by consulting a medical specialist [3]. Although, little is known about the consequences of referral non-compliance and consequences probably depend on the specific reasons for noncompliance, it could delay diagnosis and treatment and could lead to poorer health outcomes [4].

Studies on referral compliance are mainly from the United States, and show compliance rates between 63 and 83 % in the general population [3, 5, 6]. Results of these studies might not be transferrable to most European countries as the unmet care needs are generally higher in the United States, probably due to a lower level of health insurance coverage and higher levels of out-of-pocket payments [7]. In addition, most of these studies rely on the recall of patients and/or physicians. Literature on non-adherence with prescribed medication show an overestimation of self-reported compliance compared with claims data [8].

As referral compliance is important for an effective gatekeeper system, it is necessary to increase knowledge about referral compliance outside the United States. The objective of the current study was to i) estimate the referral compliance to medical specialist care in the Netherlands using claim data and ii) identify patient (predominantly socio-demographic factors) and practice characteristics that predict referral compliance.

Box 1: Health insurance coverage in the Netherlands

**Table:** 

In the Netherlands, basic health insurance is obligatory for all residents. The benefit package of the basic health insurance is defined by the government and consists among others of medical care provided by general practitioners (GPs), medical specialists and midwives, hospital care, medical aids and devices and pharmaceutical care [9]. For basic health insurance, a compulsory deductible (amount of expenses that must be paid out-of-pocket before an insurer will pay any expenses) of €150-€165 (2008–2010) is in operation for all individuals aged 18 or older. General practice care and maternity care is exempted from the deductible [9]. In other words, visiting a GP does not result in additional costs, but consulting a medical specialist may results in out-of-pocket payments if the compulsory deductible has not been met at the time of claim processing.	

## Methods

### Study design and population

This is an observational cross-sectional study analysing the association between patient and practice characteristics and the compliance with referrals of general practitioners (GPs) to medical specialist care in the Netherlands from 2008 until 2010. Combined data on referrals from 2008–2010 was used from the electronic health records (EHRs) of general practices that participated in NIVEL Primary Care Database (NIVEL PCD). Referral data includes among others information about referrals from GPs to medical specialist care with for each referral the date, the medical specialty and the diagnosis for which the patient was referred to the medical specialist. In these years combined, 140 general practices (3.4 % of general practices in the Netherlands) were included in the database. General practices are selected based on the quality of their EHR and representative of the Dutch GP population. Overall, GPs that participate in NIVEL PCD are representative of the Dutch GP population with respect to age, gender, period of settlement, region and urbanization [10]. No differences in medical treatment are found between GPs with varying degrees of EHR use [11]. NIVEL PCD database contains longitudinal data at the patient level in terms of contacts, morbidity, prescriptions and referrals, with small yearly changes in practice composition. Dutch law allows the use of EHR for research purposes under certain conditions. According to this legislation, neither obtaining informed consent from patients nor approval by a medical ethics committee is obligatory for this type of observational studies containing no directly identifiable data (Dutch Civil Law, Article 7:458). Claims data of medical specialist care (administrative data, diagnosis related groups (DRGs)) were available from the center for information of Dutch health insurers, Vektis. Vektis collects data from all health insurers which included, among others, DRGs claimed to all health insurers in the Netherlands. A DRG comprises a more or less fixed set of secondary care services, related to a specific diagnosis, at a certain price that may or may not vary between hospitals. Medical specialists submit claims for DRGs to the patient’s health insurer. Only in exceptional cases, the care is paid by the patient itself. This claims data is made available for research through Vektis. For each DRG, the start and end date, the health insurer to whom the services was claimed, the price, and as part the DRG the diagnosis, treatment and medical specialty by whom the service was claimed is known. For this study, 2008–2011 data was used.

NIVEL PCD and Vektis data were linked on patient level on the basis of postal code, gender and date of birth (probability linkage): 80 % of the patients’ referrals could be linked. Patients who could not be linked were younger (*p* < 0.01), were living closer to a medical specialist care facility (*p* < 0.01), had less often a chronic disease (*p* < 0.01) and were more often living in a deprived urban area (*p* < 0.01). The identifying information to link data was deleted after linkage to guarantee patients’ privacy. This study was part of a larger study investigating the primary-secondary care interface [12]. For this larger study, we only included data from practices that passed a number of checks regarding the quality of data on care episodes (morbidity), referrals to medical specialist care, contacts and prescriptions. Reasons for excluding practices (*n* = 140) for a specific year (non-exclusive) were (1) incomplete data on care episodes (e.g. in less than 50 % of morbidity record a diagnosis; 25 % of excluded practice years), (2) incomplete data on contact (e.g. less than 46 weeks of contact data; 30 %), (3) incomplete data on prescriptions (e.g. Anatomical Therapeutic Chemical (ATC) code in less than 85 % of the prescription; 30 %) and/or (4) incomplete data on referrals (e.g. less than one referral per week; 50 %). An additional criterion for the present study was available data on the claimed capitation fees, since this information was necessary to differentiate patients living in a deprived urban area. Three practices in the period 2008–2010 were excluded due to this extra criterion. In total, data on 48,784 referrals from 58 general practices were included in the period 2008–2010. Overall, the practices included were representative of Dutch general practices with respect to the degree of urbanisation (included practices vs. Dutch general practice; extremely urbanised: 22 vs. 18 %; strongly urbanised: 33 vs. 26 %; moderately urbanised: 17 vs. 20 %; hardly urbanised: 19 vs. 22 %; not urbanised: 9 vs. 15 %) and region (north: 13 vs. 11 %; east: 15 vs. 20 %; west: 54 vs. 48 %; south: 19 vs. 21 %), but not with respect to practice type (solo: 22 vs. 42 %; duo: 26 vs. 32 %; group/health centre: 52 vs. 26 %; overrepresentation of group practices or health centres and underrepresentation of single-handed practices).

### Measurements

#### Dependent variable: referral compliance

For all new referrals to medical specialist care, it was determined whether the patient had a claimed DRG in a half year period after the referral. We only considered DRGs claimed by a specific medical specialty to whom the patients was referred to. Referrals to psychiatry were not included as no DRG system was in operation for psychiatry during the study period.

#### Predicting variables: patient characteristics

Patient characteristics were included that may predict referral compliance and were available in the NIVEL PCD: age (categorised), gender, neighbourhood socioeconomic status (SES), morbidity and distance to the nearest medical specialist care facility. Neighbourhood SES was defined in two ways: i) living in a deprived urban area and ii) social status of the neighbourhood. Whether patients lived in a deprived urban area was based on claimed capitation fees. In the Netherlands, GPs receive higher capitation fees for patients living in an deprived urban area. GPs can claim the capitation to the patient’s health insurer, with different claim codes for patients in deprived urban areas. The deprived areas are determined on a five-digit postcode level and are based on the percentage of non-western immigrants, percentage of residents with a low-income, percentage of residents aged 15–64 years of age without a job (excluding students) and the level of urbanization [13]. Neighbourhoor social status score in 2010 was derived from The Netherlands Institute for Social Research (SCP) and are calculated on a four-digit postcode level [14]. This score reflects the social status of a neighbourhood, compared to other neighbourhoods in the Netherlands. It is a composite measure calculated from individual characteristics of neighbourhood residents, i.e. mean neighbourhood income, percentage of residents with low-income, percentage of low-educated residents, and percentage of residents without a job. Status scores were categorized in quartiles based on the present data (low, moderate, high, and very high status). Status score is a common indicator for neighbourhood SES in the Netherlands. The main difference between both indicators for neighbourhood socioeconomic status is the role of level of urbanization. Eighty-five percent of the patients living in a deprived urban area is found in the lowest quartile of the status scores. Of patients in the lowest quartile only 54 % also lived in a deprived urban area. Morbidity was defined as the number of chronic diseases in a year, and was based on a list of chronic diseases used by the Dutch National Institute for Public Health and the Environment [15]. Distance to the closest medical specialist care facility (including hospitals, outdoor departments and independent clinics) by road was assessed on the basis of postal codes centroids.

#### Predicting variables: practice characteristics

We included practice characteristics that were available in NIVEL PCD and that give an indication about the health care provided to patients, and which therefore may influence the referral compliance: average number of face-to-face contacts with GP, guideline adherence related to referrals, presence of primary care nurse and type of practice. Guideline adherence related to referrals was based on five guideline adherence indicators, in turn based on clinical guidelines described in detail elsewhere [16]. Mean adherence rates were calculated per indicator per practice per year. The average of all indicators for guidelines related to referrals was calculated per practice per year and included in the analyses. The presence of a primary care nurse was determined per practice per year. Primary care nurses are predominantly involved in care for chronically ill patients. Practice type was categorized in (1) single-handed-, (2) duo-, and (3) group practice or health centre.

### Statistical analyses

Descriptive analyses were performed to examine the patient and practice characteristics and estimate referral compliance in general and by referral diagnosis (chapters of International Classification of Primary Care, ICPC). To investigate which patient and practice characteristics are associated with referral compliance, we performed logistic multilevel regression analysis, using a model with three-levels, since the data is hierarchically structured (referrals nested within patients and patients nested within general practices, random intercept model). Multilevel analysis corrects for the cluster effect of hierarchically structured data. The two indicators for neighbourhood SES were separately inserted in the model with all other patient and practice characteristics, due to collinearity (model 1: deprived urban area, model 2: social status of the neighbourhood). All analyses were performed using MLwiN 2.30 (IGLS estimation; 1st order MQL).

## Results

### Patient and practice characteristics

Patients with a referral were more often female (58.7 %), were on average 47.6 years old and had a median distance to the nearest medical specialist care facility of 3 km (Table 1). Almost half of the referrals were for patients with a chronic disease and almost 60 % of the referrals were for patients of 45 years or older. Nine percent of the referrals were for patients who lived in a deprived urban area. The mean social status score of the neighbourhood was -0.30, which is lower than the average in the Netherlands of 0.17 in 2010. Of the general practices included, more than half of the practices was a group practice or health centre and most had a primary care nurse (86.7 %). The average number of face-to-face contacts with GPs was 2.63 contacts per patient per year, and adherence to guidelines related to referrals was in general high (90.0 %) and only varied slightly (SD:4.3).

**Table 1:** Patient and practice characteristics of patients with a referral to medical specialist care, 2008–2010, 48,784 referrals

Patient characteristics	
Gender (% female)	58.7 %
Age	
0–17 year	12.2 %
18–24 year	5.2 %
25–44 year	24.1 %
45–64 year	33.6 %
65–74 year	13.3 %
75 years or older	11.7 %
Chronic diseases	
No chronic disease	50.7 %
1 chronic disease	29.8 %
2 chronic diseases	12.3 %
3 or more chronic diseases	7.1 %
Distance to nearest medical care facility (km)	3 (1–11)
Living in deprived urban area	9.3 %
Social status of the neighbourhood	
Quartile 1: -5.9 - -0.9	22.8 %
Quartile 2: -0.9 - -0.2	27.9 %
Quartile 3: -0.2 - 0.62	23.1 %
Quartile 4: 0.62 - 2.86	26.3 %
Practice characteristics (per year)	
Practice type	
Single-handed practice	25.6 %
Duo practice	20,0 %
Group practice or health centre	54,4 %
Face-to-face contacts with GP	2.63 (0.37)
Guideline adherence referrals	90.0 (4.3)
Primary care nurse	86.7 %

### Referral compliance

Of all referrals to medical specialist care, 86.6 % (SD:34.1) were consumed. In Table 2, compliance by referral diagnosis is shown. Compliance rates varied between referral diagnoses (Chi square *p* < 0.001). Compliance was lowest for patients referred for digestive health problems (*n* = 4201; 76.5 %) and for the male genital system (*n* = 1296; 77.1 %), and was highest for patients referred for ear problems (*n* = 2498; 92.1 %) and respiratory health problems (*n* = 3535; 90.5 %). Distinguishing between referrals for symptoms (ICPC:1–29; *n* = 23,972) and for diagnosed diseases (ICPC:70–99; *n* = 23,153) showed slightly higher compliance for diseases (87.4 % vs. 86.0 %; Chi square *p* < 0.001).

**Table 2:** Referral compliance by referral diagnosis, 2008–2010^a^

	*N*	Compliance
A General and unspecified	1712	83,8 %
D Digestive	4201	76,5 %
F Eye	4185	88,7 %
H Ear	2498	92,1 %
K Circulatory	3913	88,3 %
L Musculoskeletal	10,119	87,9 %
N Neurological	2383	88,7 %
R Respiratory	3525	90,5 %
S Skin	6234	87,2 %
T Endocrine, metabolic and nutritional	1329	81,1 %
U Urology	1323	90,4 %
X Female genital system and breast	2420	90,2 %
Y Male genital system	1296	77,1 %
Other (Blood, blood forming organs, lymphatics, spleen, psychological, pregnancy, childbirth, family planning, social problems)	2086	82,7 %

^a^In 3.2 % of the referrals no referral diagnosis was available, these were excluded

### Association between patient and practice characteristics and referral compliance

Living in a deprived urban area was associated with lower referral compliance (OR:0.85; 95 % CI:0.76–0.95) (Table 3). Social status of the neighbourhood where patients were living was not associated with compliance. Patients living further away from a medical specialist care facility were more compliant, but the difference was not relevant (OR:1.01; 95 % CI:1.00–1.02). No differences in compliance were found between patients with or without chronic diseases. Patients in the age 18–44 years complied less often with referrals compared with patients aged 0–17 years, whereas patients ages 65 years and older were more compliant. None of the practice characteristics was associated with referral compliance.

**Table 3:** Logistic multilevel regression analysis on association of patient and practice characteristics with referral compliance, 2008–2010^a^

	Referral compliance	
	Model 1	Model 2
	OR (95 % CI)	OR (95 % CI)
Gender (ref: female)	0.99 (0.94–1.05)	0.99 (0.94–1.05)
Age (ref: 0–17 year)		
18–24 year	0.74 (0.65–0.84)*	0.74 (0.65–0.85)*
25–44 year	0.79 (0.72–0.87)*	0.79 (0.72–0.87)*
45–64 year	1.01 (0.92–1.11)	1.01 (0.92–1.11)
65–74 year	1.22 (1.08–1.37)*	1.22 (1.09–1.38)*
75 years or older	**1.15 (1.02–1.30)**	**1.16 (1.02–1.31)**
Chronic diseases (ref: no chronic disease)		
1 chronic disease	1.02 (0.96–1.08)	1.02 (0.96–1.08)
2 chronic diseases	1.01 (0.92–1.11)	1.01 (0.92–1.11)
3 or more chronic diseases	1.04 (0.92–1.17)	1.04 (0.92–1.17)
Distance to nearest medical specialist facility (km)	1.01 (1.00–1.02)*	1.01 (1.00–1.02)*
Livingin deprived urban area	0.85 (0.76–0.95)*	
Social status of the neighbourhood (ref Quartile 4: -0,62 -2,86)		
Quartile 1: -5.9 - -0.9		0.95 (0.86–1.04)
Quartile 2: -0.9 - -0.2		1.02 (0.93–1.11)
Quartile 3: -0.2 - 0.62		1.03 (0.93–1.14)
Practice type		
Duo practice	0.89 (0.74–1.06)	0.89 (0.73–1.07)
Group practice or health centre	1.07 (0.93–1.24)	1.07 (0.92–1.24)
Number of face-to-face contacts with GP	1.10 (0.92–1.30)	1.09 (0.91–1.30)
Guideline adherence referrals	1.00 (0.99–1.02)	1.00 (0.99–1.02)
Primary care nurse	1.11 (0.94–1.32)	1.13 (0.95–1.36)

^a^Adjusted for referral diagnosis (ICPC chapters)

*significant at the *p* = 0.01 level

bold font: significant at *p* = 0.05 level

## Discussion

Patients complied with referrals to medical specialist care in 86.6 % of the referrals. Only patient characteristics and not practice characteristics were associated with compliance: patients living in a deprived urban area and patients aged 18–44 years were less compliant, and patients aged 65 year or older were more compliant.

### Strengths and limitations

We used a large dataset with routinely recorded referrals, enabling us to analyse the association between patient and practice characteristics and referral compliance. A number of issues should be considered. Eighty percent of the patients’ referrals could be linked between NIVEL PCD and Vektis on the basis of postcode, gender and date of birth. Linkage was associated with several patient characteristics. Especially the lower number of patients living in a deprived urban area that could be linked, could have led to an overestimation of referral compliance. Referral compliance was based on a claimed DRG by a specific medical specialty to whom the patient was referred. It is possible that patients were referred to and treated by another medical care specialty within a medical specialist care facility, which may have led to an underestimation of the referral compliance. In addition, patients may have visited health care providers abroad. These services are only included in the Vektis data if the service is claimed and reimbursed by a Dutch health insurer. This is dependent on the health insurance policy, and especially the possible preferred provider policy. Further, the severity of the health problem for which the patient was referred was unknown and this may have influenced the compliance rate. Finally, the included general practices were not representative of Dutch general practices with regards to practice type. As the practice type was not (significantly) associated with referral compliance, we do not expect this to have influenced our results. A large number of practices did not meet the inclusion criteria. Although based on known characteristics of general practices we have no indication that included general practices differed from excluded general practices, included practices could differ on other factors. This could have affected our results.

### Comparison with existing literature

Compared with studies on the referral compliance in the general population mainly from the United States, compliance is slightly higher in the Netherlands (87 % vs. 63–83 %) [3, 5, 6], although comparisons are not straightforward due to differences in methods. As mentioned, most of the US studies rely on self-reported compliance, which generally give an overestimation of compliance [7]. Therefore, the difference between the Netherlands and United States is probably larger. This higher rate of compliance could be due to a lower level of health insurance coverage and higher levels of out-of-pocket payments in the United States [8].

Patients aged 0–17 years, or their parents, and patients aged 65 years and older more often complied, which was not found in studies from the United States [3, 17–19]. A reason for the differences in referral compliance with age could lay in the compulsory deductible which is not in operation for patients aged 0–17 years. For patients aged 65 years or older, an explanation could be found in the high prevalence (>70 %) of chronic diseases which is accompanied with increased health care costs [20]. For these patients, the compulsory deductible is easily met, so they may be less restrained to comply. Analysis with only chronic conditions as independent factor (and not age) showed a higher compliance rate in patients with one or more chronic conditions (1 chronic disease, OR:1.11; 2 chronic diseases, OR: 1.18; 3 or more chronic diseases, OR: 1.28), which seems to confirm this hypothesis.

Patients living in a deprived urban area less often complied. Previous research on the uptake and participation in physical activity referral scheme in the United Kingdom, also showed less compliance in patient living in more deprived neighbourhoods [21]. However, we did not find an association between neighbourhood social status and referral compliance. The main difference between both neighbourhood SES indicators is the role of level of urbanization, which is not included in the neighbourhood social status. So, it seems that especially patients in deprived urban areas and not generally deprived areas less often comply. Further qualitative research is needed to gain more insight in the true causes of non-compliance in these areas. A large literature review on non-adherence with prescribed medication shows that non-adherence is a summary effect of multiple determinants, and that many socio demographic factors have an inconsistent impact on adherence when comparing adherence studies [22]. Patient attitudes and beliefs in favour of diagnosis, medication and health recommendations, and patient’s self-efficacy do tend to be related with non-adherence. It may be that the lower referral compliance rate in patients living in a deprived urban area be explained by differences in patient attitude and health beliefs.

Characteristics of general practices were not associated with referral compliance. Patients of GPs who complied more to the guidelines about referrals did not comply more often and also practice type and the number of contacts were not associated with compliance. The number of general practices included was relatively limited, which might explain non-significant differences in the case of the number of contacts and presence of a primary care nurse. Previous research showed that a longer relationship between patient and physician was associated with increased referral compliance [3]. Continuity of care is highly valued in Dutch general practice in general, which might explain why we did not find differences with practice characteristics [23]. On the other hand, the included practice characteristics might not be suitable indicators to explain differences in referral compliance, as for example we did not included indicators about the communication style of GPs, which has shown to have an impact on adherence with prescribed medication [22].

## Conclusions

For the gatekeeper system to be effective it is vital that patients who are referred comply with the referral by attending a medical specialist [3]. Our study showed that 13 % of the patients did not use their referral. Patients can have various reasons not to comply, such as the belief that the health problem has resolved, lack of time or long waiting times [3, 24, 25]. Or patients may visit alternative health care providers instead. The consequences of non-compliance are unknown, but a study from the United States shows that non-compliance might result in delayed diagnosis and treatment, and poorer health outcomes [4]. As the level of health insurance coverage is lower and level of out-of-pocket payments are higher in the United Stated, consequences of non-compliance might be different [7]. In addition, patients in the Netherlands are also referred for diagnostic reasons without a known underlying disease. We do not believe that the 13 % non-compliance rate can be attributed fully to health problems that have been resolved or patients that visited alternative health care providers. Further research is necessary to identify the reasons and consequences of noncompliance. However, we think a substantial part of the non-compliant patients probably do not receive adequate care. The results of this study may be used to make general practitioners more aware that some patients are more likely to be non-compliant. One way to reduce non-compliance, is to schedule appointments with a medical specialist for patients. Previous studies have shown that scheduling an appointment with a medical specialist has a strong positive effect on referral compliance [3, 20]. Another result of importance is the lower referral compliance in patients living in deprived urban areas. This may be due to lower levels of health literacy in these areas. Lower health literacy has been shown to be negatively associated with adherence with prescribed medications [22]. In addition, previous research has shown that patients with a lower income or in low income areas more often refrain from health care when faced with out-of-pocket payments [26]. If indeed these patients refrain from health care due to the compulsory deductible this might be undesirable. Further research is needed to assess the role of the compulsory deductible and health literacy in compliance with referrals.
